# A brief treatment for veterans with PTSD: an open-label case-series study

**DOI:** 10.3389/fpsyt.2023.1260175

**Published:** 2023-10-19

**Authors:** Merel Kindt, Marieke Soeter

**Affiliations:** ^1^Department of Clinical Psychology, University of Amsterdam, Amsterdam, Netherlands; ^2^Work Health Technology, The Netherlands Organization for Applied Scientific Research TNO, Leiden, Netherlands

**Keywords:** reconsolidation, imagery rescripting, trauma memory, treatment, veterans, PTSD

## Abstract

**Introduction:**

Despite the positive outcomes observed in numerous individuals undergoing trauma-focused psychotherapy for PTSD, veterans with this condition experience notably diminished advantages from such therapeutic interventions in comparison to non-military populations.

**Methods:**

In a preliminary study we investigated the efficacy of an innovative treatment approach in a small sample of veterans (*n* = 7). Recognizing that accessing and targeting trauma memory in veterans with PTSD may be more challenging compared to other patient populations, we employed unique and personalized retrieval cues that engaged multiple senses and were connected to the context of their trauma. This was followed by a session focused on memory reconsolidation, which incorporated both psychological techniques (i.e., imagery rescripting) and a pharmacological component (i.e., 40 mg of propranolol).

**Results:**

The findings from this small-scale case series cautiously indicate that this brief intervention, typically consisting of only one or two treatment sessions, shows promise in producing significant effects on symptoms of PTSD, distress and quality of life.This is particularly noteworthy given the complex symptomatology experienced by the veterans in this study.

**Conclusion:**

To summarize, there are grounds for optimism regarding this brief treatment of combat-related PTSD. It appears that the potential for positive outcomes is far greater than commonly believed, as demonstrated by the encouraging results of this pilot study.

## 1. Introduction

The concept that soldiers are tormented by the haunting memories of their wartime ordeals is a recurring theme that echoes through the ages, from the epic verses of Homer’s Iliad, the poetic prose of Shakespeare to the poignant pages of Tolstoy’s ‘war and peace’. Soldiers who were exposed to the trauma of war often experienced a range of symptoms, including anxiety, depression, nightmares, flashbacks, and physical symptoms such as shaking and trembling. Labeling these symptoms as the shell shock syndrome in military personnel during World War I was a significant milestone in the recognition of trauma and its effects on mental health. Initially, these symptoms were often dismissed as signs of weakness or cowardice, and soldiers were sometimes even accused of faking their symptoms to avoid combat. The recognition of shell shock and the subsequent establishment of PTSD as a diagnosis in 1980 (DSM-II) has spurred the creation of effective treatments that have helped many individuals attain recovery and lead satisfying lives. Still, PTSD in past and present members of the military tends to be a chronic condition with prevalence rates varying between 3 and 17% (1–3). Around 80% of those with a diagnosis of PTSD also meets criteria for another mental health condition such as depression, substance use disorder, or another anxiety disorder (4). While trauma-focused psychotherapy^1^ may yield positive results for many individuals suffering from PTSD, veterans with PTSD benefit significantly less than non-military populations (5–8). After receiving trauma-focused psychotherapy for combat-related PTSD, approximately two-thirds of veterans still experience the lingering consequences of this disorder (7, 9). Another challenge arises as a considerable number of veterans prematurely end their treatment with dropout rates ranging from 25 to 48% (9, 10). Notwithstanding the advances in the field, it is increasingly evident that PTSD continues to be a complex and daunting condition for military personnel and veterans, posing formidable obstacles for traditional trauma-focused psychotherapies. Here, we examine the effectiveness of a novel treatment approach with the goal of mitigating the problem of treatment resistance and the high rates of dropout commonly observed in veterans.

Prior to delving into the details of our innovative treatment approach, we propose several plausible explanations that could clarify the relatively lower effectiveness of trauma-focused interventions in treating veterans with PTSD. Considering that PTSD is viewed as a disturbance of emotional memory (e.g., 11, 12), most psychotherapeutic approaches concentrate on exploring and addressing the individual’s recollection of the traumatic event or its meaning. Regardless of the specific therapeutic approach employed, they all involve revisiting the most distressing and agonizing memories associated with the traumatic experience. These memories, commonly referred to as “hotspot memories” and identified through the intrusive symptoms they elicit, serve as the basis for therapy (13–17). Successful reliving of these memories requires a focus on the sensory details and emotional responses that are integral to the memory (12, 18, 19). While perceptual memory reactivation may not be essential for reducing symptoms, it may facilitate a shift in meaning that ultimately predicts improved treatment outcomes (20). The general procedure to reactivate trauma memory is to instruct patients to imagine or describe the most disturbing traumatic situations, or the situation that is related to their hotspot memories. Yet, deliberately reliving the battlefield within a therapeutic context does not always guarantee easy access to the emotional intensity of the experience. We must not overlook the fact that military personnel undergo extensive training to equip them with the necessary skills to withstand the arduous physical and mental challenges inherent in their duties. Since emotions such as fear, anger, and sadness can have a significant impact on decision-making and performance in high-stress situations, military training often includes instruction on how to regulate and control their emotions while on duty. Hence, the efficacy of established trauma-focused therapies in evoking a deeply moving emotional response when recalling trauma memories in veterans might pose greater challenges compared to non-military individuals with PTSD. Pinpointing the per-symptom effectiveness of treatments in uncontrolled clinical settings in Israel indeed showed that only a small number of veterans (15.8%, n = 709) experienced minimal relief from symptoms of intrusive traumatic reexperiencing, while two other mnemonic symptoms, namely flashbacks and inability to recall an important aspect of the trauma, exhibited no response to treatment at all in this group of veterans (21). It is important to mention that not all veterans received trauma-focused therapy in this study. Nonetheless, the failure to effectively address these mnemonic symptoms in veterans may perpetuate other features of the disorder as well (21).

Another potential limitation is that current therapies were originally designed to address the excessive fear responses to trauma reminders, rather than the multifaceted emotions that may be present in military-related PTSD. Feelings of sadness, anger, shame, guilt, a sense of powerlessness, and betrayal are all common experiences that people may experience when being exposed to trauma (22). These feelings are particularly intense for members of the military who are often confronted with demanding ethical or moral decisions during their service (23). While decision-making is often likely to be consistent with their military codes of conduct, substantial levels of psychological distress can still be experienced when they perpetrate, witness or fail to prevent actions that run counter to their core moral or ethical values (24). This elevated state of distress is identified as ‘moral injury’, a condition closely linked to feelings of guilt, anger and shame (25, 26). As a result, individuals with combat-related trauma may not fully benefit from existing treatments, as their emotional needs remain unaddressed. When it comes to addressing the needs of military personnel dealing with PTSD, it’s also essential to consider the emotional toll that therapy can take on veterans. Most trauma-focused therapies require a prolonged course of treatment, which can be emotionally very taxing for military personnel. It is promising though that a recent study has shown a correlation between massed exposure therapy, which consists of delivering a full treatment program over a shorter duration, and a decrease in the number of veterans who discontinue the treatment (9). Hence, to truly develop more comprehensive and effective treatments for military-related PTSD, further research is necessary to better understand the emotional needs of veterans.

As an initial attempt to overcome the limitations of existing treatments for PTSD, we have developed a novel and concise intervention tailored to combat-related trauma. While most therapies rely on visual and verbal retrieval cues to access emotional memory, we believe that the use of multi-sensory and context-related stimuli as retrieval aids has been surprisingly underutilized. For instance, odors similar to the trauma context could be particularly powerful cues to spontaneously evoke autobiographical memories with a strong emotional resonance (27–31). One advantage of utilizing odors as retrieval cues is that the brain regions involved in olfaction have direct connections to the amygdala and entorhinal cortex (32, 33), both of which are involved in emotion processing and memory. To enhance access to emotional memory, we developed idiosyncratic virtual reality worlds that incorporate multi-sensory retrieval cues, including 3D visual, auditory, olfactory, and bodily information, tailored as much as possible to everyone’s personal experiences. After this brief memory reactivation procedure (i.e., 2 min), the focus was shifted toward the idiosyncratic memory representation of the veteran. On the basis of information provided in the intake we had formulated hypothesized stuck points that we tried to directly target in treatment. This part consisted of imaginal exposure combined with rescripting with the rationale that new perspectives on what happened during trauma are most effectively achieved by experiencing new views and emotions which were not possible at the time of the trauma (17, 34, 35). In a previous randomized-controlled trial in nonmilitary-related PTSD, we demonstrated that the addition of imagery rescripting to imaginary exposure led to a significant reduction of treatment dropouts, and better effects on guilt, anger and shame as compared to exposure alone (34).

Finally, a pivotal modification in the current intervention was to utilize the process of memory reconsolidation as an alternative means of inducing change. Most trauma-focused therapies are rooted on extinction learning with the notable limitation that it can only eliminate the fearful responding while leaving the original trauma memory intact (36, 37). As a consequence, the intact trauma memory may resurface thereby explaining the relatively high relapse rates even after initial treatment success (38, 39). In contrast, the hypothesis of memory reconsolidation suggests that it may be possible to target the trauma memory directly, with the promise of an instantaneous and more persistent alleviation of symptoms. Memory reconsolidation refers to the process that upon memory retrieval, items in long-term memory may temporarily return into a labile state requiring *de novo* protein synthesis in order to persist (40). This cascade of neurobiological processes offers a window of opportunity for targeting fear memories with amnestic agents. The crucial role of central noradrenergic signaling in the process cascade (41) suggests that the β-adrenergic blocker propranolol is a viable option for effectively interfering with memory reconsolidation in humans. Indeed, preclinical research has compellingly shown that β-adrenergic blockade during reconsolidation can disrupt fear memories in healthy individuals (e.g., (42); see for a review (43)) and in people with a fear of spiders ((44, 45); but see (46)). Even though these findings point to a revolutionary new treatment for emotional memory disorders, the success of reconsolidation interventions is not guaranteed (47). The effect of the intervention depends on whether memory retrieval effectively triggers reconsolidation. Only if the retrieval experience contains novel or unexpected information (i.e., prediction error), the memory engram will be destabilized (48, 49). While clinical research in patients with PTSD initially revealed a reduction in fear responding following a reconsolidation intervention (17, 50–52), these findings could not always be replicated in several follow-up trials ((53); see for a review (54)). It is worth highlighting that the design of these previous clinical trials raises several questions with respect to the necessary conditions for a reconsolidation intervention. The effectiveness of this intervention hinges on two key conditions: (i) the retrieval procedure should lead to the destabilization of the trauma memory, and (ii) the amnestic drug should interfere with the subsequent reconsolidation of that fear memory (43). In previous clinical trials, script-driven imagery was employed to reactivate traumatic memories, despite the fact that this method was explicitly designed to assess the passive retrieval of these memories, rather than their reconsolidation. Additionally, the timing of drug administration in these clinical trials (specifically, the use of long-acting propranolol after the reactivation of the traumatic memory) does not align with the reconsolidation hypothesis. We carried out a series of experiments aimed at exploring the optimal timing for administering propranolol. Our findings revealed a rather narrow temporal window, spanning less than four hours following memory reactivation, during which the β-adrenergic receptors assume a pivotal role in the reconsolidation of fear memories (47, 55).In summary, unlike traditional trauma-focused interventions that require multiple sessions with gradual and often temporary improvements, the memory reconsolidation intervention (i.e., Memrec) offers an unique approach to treating combat-related PTSD by providing a single, possibly effective treatment session that results in a sudden reduction in emotional symptoms. This innovative treatment approach also represents a departure from the conventional use of pharmaceutical agents to alleviate PTSD symptoms, as it involves a one-time administration of a very common drug (i.e., 40 mg propranolol HCl) after reliving and rescripting the combat-related trauma memory. In the current case series involving seven veterans we tested the effectiveness of Memrec for trauma in which we aimed to address the unparalleled complexities that are typically associated with treating combat-related PTSD.

## 2. Materials and methods

### 2.1. Participants

Participants were combat-exposed Dutch military veterans referred for treatment at ARQ Centrum ‘45, the Dutch national center for diagnostics and treatment of patients with long-lasting trauma-related disorders. Inclusion criteria were (a) aged between 18–65 years, and (b) a diagnosis of PTSD based on the Clinician-Administered PTSD Scale (CAPS-5) (56). The exclusion criteria included (a) any other relevant treatment for PTSD within 3 months before the start of the study, (b) start of new psychotropic medication within 3 months before the start of the study – medication used for longer periods could be continued, (c) life-time psychosis, (d) acute suicide risk, and (e) any contra-indications for the use of propranolol. All participants gave written informed consent, and the protocol was approved by the Medical-Ethical Committee of the Amsterdam UMC.

### 2.2. Outcome measures

The following measures were completed pre-treatment, as well as 1-month and 3-months post-intervention.

#### 2.2.1. PTSD checklist for DSM-5

The PCL-5 is a 20-item self-report measures that assesses the 20 DSM-5 symptoms of PTSD (57), and can be used to monitor symptom change, to screen for PTSD, or to make a provisional PTSD diagnosis. Respondents rate each item from 0 = “not at all” to 4 = “extremely” to indicate the degree to which they have been bothered by that particular symptom over the past month. A total symptom severity score can be obtained by summing the scores for each of the 20 items, range = 0–80. DSM-5 symptom cluster severity scores can be obtained by summing the scores for the items within a given cluster, i.e., cluster B = items 1–5, cluster C = items 6–7, cluster D = items 8–14, and cluster E = items 15–20. A PCL-5 cut-off score between 31–33 is considered indicative of PTSD (58). Evidence suggests that a 15–20 point change represents a clinically significant change (59). The PCL-5 is a psychometrically sound instrument that can be used effectively with veterans (58, 60). An additional item was added to the PCL-5 to ask about distress and interference caused by PTSD symptoms on a 5-point scale of frequency and severity ranging from 0 = “not at all” to 4 = “6 or more times a week” (61).

#### 2.2.2. Beck Depression Inventory - Second Edition

The BDI-II is a widely used 21-item self-report inventory measuring the severity of depression over the previous 2 weeks (62). The items are rated on a 4-point severity scale and are summed to give a total score, with a range of 0–63. A higher score on the BDI-II denotes more severe depression, with norms of 0–13 = minimal depression, 14–19 = mild depression, 20–28 = moderate depression, and 29–63 = severe depression. The BDI-II is considered a valid and reliable instrument (63), and is often used as outcome instrument in treatment research.

#### 2.2.3. Mental Health Quality of Life Questionnaire

The MHQoL is a self-report questionnaire that captures and values 7 dimensions relevant to the quality of life of people with mental health problems: i.e. self-image, independence, mood, relationships, daily activities, physical health, and future. The MHQoL comprises seven questions each with four response levels, ranging from “very satisfied” to “very dissatisfied.” The MHQoL index score can vary from 0 to 21, with higher scores indicating better quality of life. The MHQoL demonstrates favorable psychometric properties, and shows promise as a simple and effective measure to assess quality of life in people with mental health problems (64).

### 2.3. General treatment procedure

The general procedure of the Memrec-intervention for veterans with PTSD involved a screening session, an intake session, one or two intervention sessions, and a series of three assessment sessions. During screening, the study procedures were explained, patients provided written informed consent, and the baseline assessment was completed – t0, see below. Next, a medical screening was conducted to rule out any possible contraindications for the use of propranolol, including blood pressure < 90/60 mmHg, heart rate < 60 bpm, a range of adverse medical conditions such as heart disease or asthma, and the use of other medications that may negatively interact with propranolol.

In the intake session, trauma memory was explored. Specifically, the patients’ most painful trauma memories were identified by using the contents of their intrusive symptoms as a guide. In the intervention session, these “hot spots” (13, 16) subsequently served as the focus of the reactivation procedure. Also, patients’ safety behaviors were identified (e.g., distracting oneself), and they were instructed to drop these behaviors during reactivation.

Given the potential of multi-sensory input during treatment to increase engagement and support activation of traumatic memories (65), the reactivation procedure started with experiencing war in a “sensory-reality” application^2^, in which audio-visual experiences are synchronized with scent, temperature, air flow, and tremble. After the ±2-min film displaying relevant war images^3^, the patients were instructed to close their eyes and reactivation focused on their personal hotspots. As the patients revisited the most challenging aspects of the traumatic event, they were encouraged to envision how they wished they had responded during the trauma, aiming to address instances of moral injury they had experienced. A single dose of 40 mg of propranolol was administered immediately upon successful rescripting of the combat-related memory, which was evidenced by patients’ self-report and therapist observations.^4^ Next, a 120-min waiting period followed to monitor the intended effects of propranolol on autonomic responding: blood pressure and heart rate were recorded at the beginning and end of the intervention-session.

A week after the intervention, trauma memory was again relived through both the sensory-reality pod and imaginal exposure. If patients reported no meaningful reduction of their PTSD symptoms and still showed high-end distress ratings during imaginal reliving, they were offered a second pill intake, and an extra post-intervention session followed a week later. This was repeated only once.

The assessment sessions took place at screening (t0), at 1-month (t1) and 3-months (t2) post-treatment. Assessments included the PCL-5 (57) as the primary outcome, and the BDI-II (62), and MHQoL (64) as secondary outcomes. Moreover, at these timepoints, patients were interviewed about any changes brought about by the Memrec-intervention.

## 3. Results

Here, we present seven cases from an open-label pilot study of the reconsolidation intervention for veterans with PTSD. The patients were on the waiting list for trauma-focused treatment at ARQ Centrum ‘45, when offered the possibility of this intervention. All cases were treated by the first author (MK), who is a registered healthcare psychologist and an experienced cognitive behavioral therapist.

### 3.1. Case descriptions

#### 3.1.1. Case conceptualization_1

Case 1 concerns a 47-year old veteran who had been deployed to former Yugoslavia in 1996. His PTSD symptoms developed progressively since 2001, after watching a documentary on the fall of Srebrenica. His most prominent symptoms included flashbacks alternating with amnesia of a traumatic night during the time of deployment, and being hyper-alert in public places. He had received three sessions of EMDR, without any symptom relief.

The treatment focused on that specific night, which was pitch-dark and full of explosions. While lying on his stomach in the dirt, threats were made over the radio: “fuck you IFOR, we are gonna get you.” He experienced intense fear, and eventually blacked-out: he cannot remember part of the night and is afraid he emptied his weapon, which will not let him go. With the imagery rescripting, we aimed at enabling him to tolerate the worst-case scenario: i.e. him shooting his gun. Even though overwhelmed by this, it unexpectedly felt as a relief to be able to do what he feared most had happened. He experienced intense emotions, and at several moments he cried. 40 mg of propranolol was given after reactivation.

**Figure 1:**
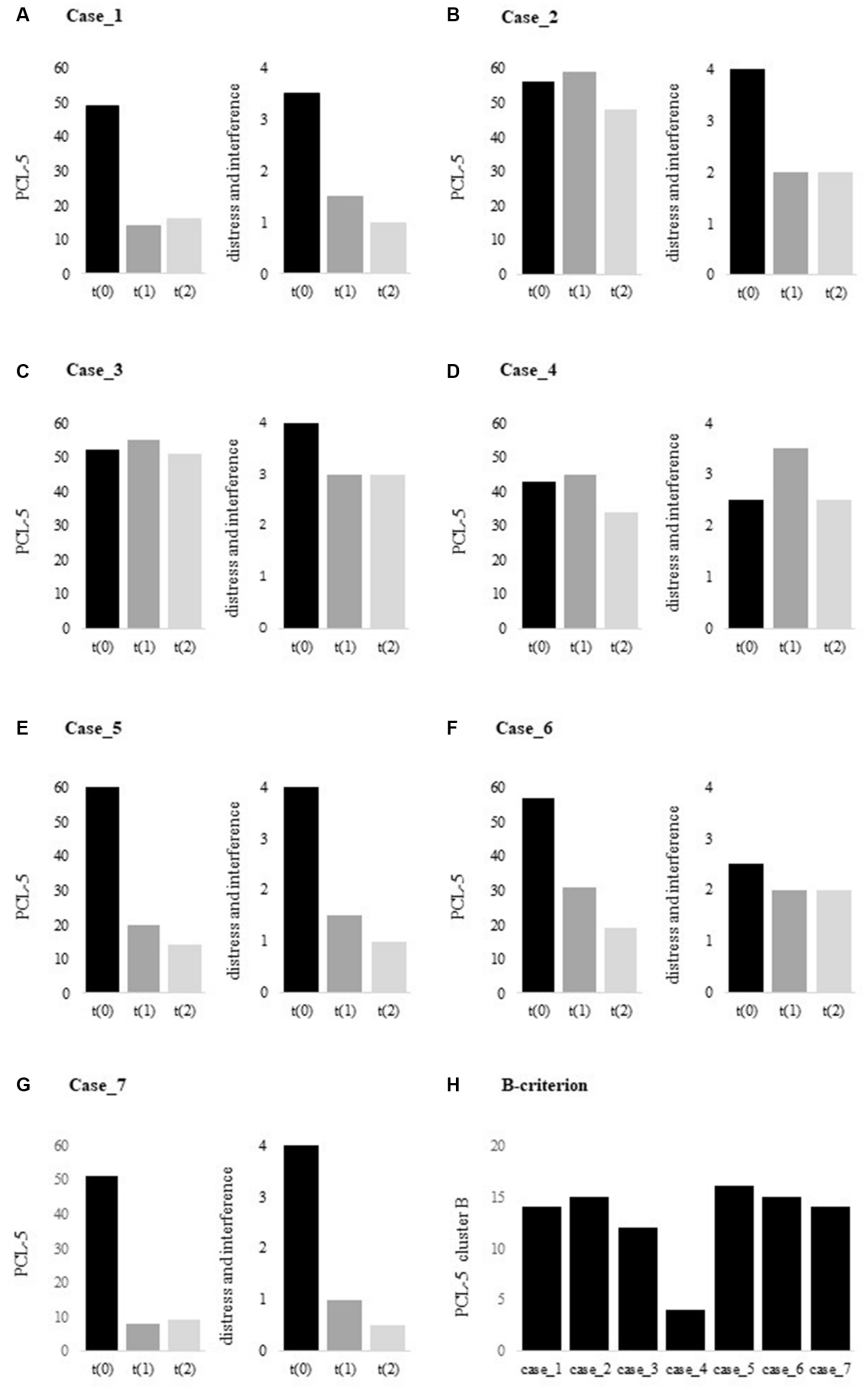
**(A–G)**. PCL-5 total scores at t(0), t(1) and t(2), and **(H)**. PCL-5 cluster B scores at t(0). PCL-5, PTSD Checklist for DSM-5 [range 0–80].

A week later, re-experiencing the traumatic night no longer triggered any fear or anxiety, and he had stopped worrying about what might have had happened. After a month, he hardly ever thought about the particular night anymore, and his nightmares had disappeared. He felt less alert, and was now able to go to grocery stores without scanning for potential danger. His improvements remained stable over time, as reflected in (i) a drop of >30 points on the PCL-5 and (ii) his scores on the secondary measures. He no longer met the criteria for a PTSD diagnosis – see Figure 1A and Table 1.

**Table 1:** Patients’ total scores on the BDI and MHQoL at t(0), t (1) and t (2).

	t(0) - baseline	t (1) – 1-month FU	t (2) – 3-months FU
**Case_1**			
BDI	29	8	7
MHQoL	6	14	13
**Case_2**			
BDI	36	29	28
MHQoL	5	11	13
**Case_3**			
BDI	26	22	25
MHQoL	5	9	10
**Case_4**			
BDI	31	25	24
MHQoL	9	8	7
**Case_5**			
BDI	17	12	4
MHQoL	12	16	16
**Case_6**			
BDI	21	30	21
MHQoL	6	6	7
**Case_7**			
BDI	23	6	11
MHQoL	13	16	19

BDI, Beck Depression Inventory [range 0–63]; MHQoL, Mental Health Quality of Life Questionnaire [range 0–21].

#### 3.1.2. Case conceptualization_2

Case 2 is a 39-year old veteran who served in Afghanistan. He suffered from severe PTSD, with nightmares, flashbacks, feeling hyper-alert, and panic attacks in public places. He previously received pharmacological treatment for depression, with sufficient symptom relief. Ultra-long distance trail running offered him an outlet for releasing some of his tension and anxiety.

Here, the treatment focused on two intrusive memories where he had felt powerless: (i) a suicide attack on a market with many civilian casualties where he was unable to help, and (ii) the moment a Dutch army vehicle was struck by a roadside bomb and he was not allowed to fight. With the imagery rescripting, we aimed at inducing a feeling of being in control, i.e., contrary to what he had actually experienced. He was instructed to relive the moments and imagine his desired response, which triggered intense emotional feelings. Afterwards, 40 mg of propranolol was administered.

From the following day onwards, he experienced a breakthrough in emotions. He noticed that it was easier to talk about his traumatic experiences and to share his feelings. He constantly started challenging himself by going to public places such as grocery stores. Although this felt as a victory to him, it was also very distressing, which may be reflected in the PCL-5: still high overall scores at t5 but less interference in everyday life – see Figure 1B and Table 1. Indeed, he experienced an improvement in quality of life and reported being optimistic about the future, where he wants to help other veterans with PTSD through organizing run clinics and events.

#### 3.1.3. Case conceptualization_3

Case 3 concerns a 37-year old veteran who had been deployed to Iraq and Afghanistan between 2002–2011. His PTSD symptoms had developed over several years, including severe irritability or aggressive behavior, disturbed sleep, nightmares, flashbacks, and avoiding distressing memories, thoughts and feelings associated with his missions. He was diagnosed with ADHD as an adult, for which no medication. After a period of misuse of anabolic steroids in the past, he had started weekly injections of testosterone years ago to replace what his body no longer produced. He had received several non-trauma focused treatments as well as one session of EMDR, without sufficient symptom relief.

Given his diverse traumatic experiences, this treatment included two sessions followed by the intake of a pill of 40 mg propranolol. In the first session, imagery rescripting focused on an armed home invasion in Iraq, involving a playing child. We aimed at targeting his feelings of guilt by saving the child in the pertinent situation, instead of leaving her behind. During the second intervention-session, he relived the moment he was forced to leave Afghanistan due to an injury. Here we aimed at targeting his feelings of inadequacy of leaving behind his fellow soldiers as a result of his physical injuries.Throughout both interventions-sessions, he experienced intense emotions that previously he had been able to suppress.

After the Memrec-sessions, he reported to have experienced a breakthrough in trauma processing. He started to remember new events from his time of deployment, felt and shared repressed emotions, and noticed that it was easier to talk about his experiences. Although this was perceived as positive to him, his PTSD symptom severity score slightly increased – see Figure 1C. He still suffered from disrupted sleep, but note that this may also be a side-effect of the testosterone therapy (66). Despite all this, his aggressive outbursts had reduced to a minimum and he had opened up to life again, undertaking social activities with friends – see Table 1.

#### 3.1.4. Case conceptualizaiton_4

Case 4 is a 50-year old veteran who had been deployed to Bosnia and Afghanistan, and is still active in the army. He had suffered from emotional numbness since 1997, when two soldiers died under his command. He experienced profound guilt, a distorted sense of responsibility and failure. He previously received pharmacological treatment for depression, without sufficient symptom relief.

This treatment included two intervention-sessions. Despite both sessions having triggered intense emotional feelings while reliving the traumatic event(s), the intervention failed to improve the patient’s symptoms – see Figure 1D and Table 1. Indeed, he reported no change in mood or mental state. Interestingly, inspecting the PCL-5 cluster scores revealed that he showed little combat-related intrusions – see Figure 1H: cluster B score of 4, with a range of 0–20. While PTSD is a complex disorder with a broad range of other negative emotions such as guilt or anhedonia – which in fact dominated this patient’s clinical picture. Memrec is exclusively designed to target unduly intense fear, which may explain the lack of effect for this particular case.

#### 3.1.5. Case conceptualization_5

Case 5 concerns a 30-year old veteran who served in the Belgium army, and was deployed to Afghanistan in 2012. His PTSD symptoms had worsened since 2020, including nightmares, flashbacks, feeling hyper-alert, panic attacks in certain situations, a short temper or aggressive behavior, and emotional flattening and loss of interest. He had received 21 sessions of EMDR, without any symptom relief.

As part of the Quick Response Team, he had to guard the gate of an army base in Kabul, and to bring in the dead and wounded. With the first imagery rescripting session, we aimed to let him relive his emotions when sitting in front of the gate with a severely injured Afghan girl in his arms, who eventually died. Even though he was not able to picture the situation, some grief and anger surfaced, and he was administered a pill of 40 mg of propranolol. A week later, the second intervention-session focused on the situation he had to carry in coffins with corpses through the gate. This triggered intense emotional feelings, and all of a sudden he was able to vividly remember what the gate had looked like – an image of which he had completely forgotten. Again, 40 mg of propranolol was given afterwards.

After the second Memrec-session, the flashbacks and nightmares had disappeared. He felt less alert, but cheerful and happy – as was confirmed by his girlfriend. He no longer experienced panic attacks and reported it was much easier to hold his temper when frustrated, which made him feel like a better father to his one-year old son. He started living life again, and reintegrated into work. Even though he somewhat feared relapsing into PTSD, the effect persisted and improved even more over time – see Figure 1E; Table 1.

#### 3.1.6. Case conceptualization_6

Case 6 is a 49-year old veteran who served in Bosnia at the time of the fall of Srebrenica. He had suppressed the painful memories and feelings for years, but lately the impacts of his trauma surfaced. He had become very emotional, startled easily, and experienced frequent panic attacks as well as severe nightmares every single night.He had not received prior treatment.

He reacted very strongly to the sensory-reality exposure, and throughout the intervention-session he was continuously in tears. With the imagery rescripting we aimed to target two intrusive hotspots: (i) leaving behind a Danish soldier who was shot in the head – he imagined apologizing to him, and (ii) the moment he had wanted to shoot an approaching enemy vehicle, but was stopped by a fellow soldier – which had made him full of guilt and regret. He was instructed to relive this moment and imagine following his impulse: shoot and become angry at the enemy. Afterwards, 40 mg of propranolol was administered.

A week later, he reported to have experienced a positive effect of the Memrec-session: he noticed it was easier to talk about his encounters in war, and was no longer overwhelmed by emotions. His panic had suddenly disappeared as did his nightmares: he never had a “nocturnal visit” from the Danish soldier again. He had always felt excessively guilty, but had let go of this feeling entirely. And even though the news about the war in Ukraine touched him deeply and made him feel somewhat depressed, his PTSD symptoms further improved over the 3-months follow-up period – see BDI-scores in Table 1 and Figure 1F.

#### 3.1.7. Case conceptualization_7

Case 7 concerns a 44-year old veteran who had been deployed to Bosnia and Afghanistan as a vehicle recovery expert. His most prominent PTSD symptoms included recurrent nightmares, flashbacks, hypervigilance, severe irritability, and verbally aggressive behavior. He had received two sessions of exposure therapy, without any symptom relief.

The treatment focused on a hotspot memory of a nighttime firefight in Afghanistan, where two Dutch soldiers died of friendly fire and his sergeant froze with fear in the midst of the fight. He had no choice but to use a gun to survive, while – as a vehicle recovery operator – he was not trained for it. With the imagery rescripting we aimed to allow feelings of anger toward his sergeant for failing him, which he verbally acted out and, in turn, made him very emotional. Afterwards, he received 40 mg of propranolol.

A week later, he entered cheerfully. He reported that his nightmares disappeared, he had felt less alert, and that his once-frequent aggressive outbursts had reduced to zero, which was noticed by his wife and daughter. His remarkable progress remained steady, as reflected in (i) a drop of >40 points on the PCL-5 and (ii) his scores on the secondary measures – see Figure 1G and Table 1. He no longer met the criteria for a PTSD diagnosis.

## 4. Discussion

The findings of this small-scale case series cautiously suggest that Memrec holds promise for producing notable effects in just one or two treatment sessions. Clearly, four out of the seven veterans experienced a remarkable reduction in their PTSD symptoms. As for the remaining three veterans, two displayed improvements in their daily distress levels, notwithstanding the absence of a reduction on the PTSD symptom scale. Only one veteran did not exhibit any progress in his PTSD and distress symptoms, but this veteran (i.e., case 4) primarily struggled with depression rather than combat-related intrusive memories (see Figure 1H). Although the improvement in depression may appear less convincing based on these case series, three veterans showed a significant reduction in their depressive symptoms. Also, the quality of life saw a remarkable increase in most of the treated veterans (i.e., five out of the seven). It is important to note though that the long-term durability of these initial positive results remains uncertain, given that the last assessment occurred only three months after the treatment session. Nevertheless, the current findings demonstrate a general positive effect of this limited intervention, which is particularly noteworthy considering the complexity of symptoms experienced by this sample of veterans. In summary, there is reason to be optimistic for the treatment of combated-related PTSD, as it appears that there is much greater potential than commonly believed.

Certainly, we cannot conclusively attribute these effects to disrupting the process of memory reconsolidation (i.e., Memrec). Since we did not control for placebo or other trauma-focused treatments, nor for the well-known effects of a waiting list (67), the symptom reduction may also be due to other factors, including imagery rescripting, which is a fast-rising therapeutic technique yielding remarkably positive outcomes. Imagery rescripting is generally applied to treat symptoms associated with aversive mental images and is currently considered among the most effective treatments for patients with PTSD (68–71). Nevertheless, it is worth noting that the majority of these seven veterans seemed to be treatment resistant as they had already tried various trauma-focused treatments without any success, which at least suggests the presence of some specific active components in the current approach. The Memrec intervention was specifically designed to target trauma memories and it is therefore not surprising that one veteran who did not suffer from combat-related intrusions did not respond to the intervention. But also for the veterans who benefited from this intervention, it is evident that this treatment alone is not sufficient. Most participants (i.e., *n* = 6) were prior military service members with military-related PTSD, who had comorbid psychiatric conditions, had experienced multiple combat-related traumatic events, and had significant ongoing life stressors. They also grappled with profound relationship and familial challenges. Hence, there are so many other factors at play in post-war trauma survivors’ lives that Memrec or similar trauma-focused treatments should be offered as a module alongside existing treatment approaches.

Treating veterans with PTSD through memory-modifying approaches such as Memrec and Imagery Rescripting does, however, give rise to ethical considerations. These concerns encompass a range of potential issues, including the potential loss of autobiographical memories, the creation of false memories, and the unforeseen repercussions of modifying fear responses associated with traumatic memories (72). Our research findings indicate that the likelihood of losing explicit, declarative, or autobiographical memory through Memrec is very low. Individuals retained conscious awareness of previously acquired information even as the learned fear response was neutralized (20, 43). In contrast, overwhelming emotional memories can impede a patient’s ability to recall and make sense of their experiences (13). Consequently, reducing the emotional intensity of strongly encoded emotional memories through reconsolidation-based methods might paradoxically facilitate controlled recollection and integration into a coherent personal narrative, as observed in some veterans in our study (i.e., cases 5 and 6). Rather than inducing amnesia, the administration of propranolol after memory reactivation appears to primarily reduce the intense emotional impact associated with traumatic memories. Similarly, imagery rescripting may help patients reinterpret troubling past events, thereby lessening the impact of distressing memories, but it does not have the ability to erase or alter the original trauma memory (73). Another ethical concern is that some degree of fear and stress may serve a beneficial role in combat situations, preventing impulsive decisions and preparing the body for action. Hence, it may not be desirable to interfere with these emotional responses for military purposes. Nevertheless, experiments conducted in our laboratory have shown that even after mitigating learned fear through reconsolidation interventions, individuals can readily reacquire the same fear response when exposed to new threats (74). This demonstrates that military personnel who have undergone a memory-modifying intervention are not exempt from developing new emotional responses when reintroduced to real-life battlefield hazards. A clear strength of this study is its use of distinctive multi-sensoric retrieval cues to access the trauma-memory. Still, the 3D films alongside the smells were far from optimal as they could be more idiosyncratic. In addition to advance these more technological aspects of the intervention, for a next step we should systematically assess and explore the stuck points and explicitly formulate hypotheses on where and how to intervene. The current study is also limited by a small sample size, the absence of a control group, the absence of CAPS-5 assessments during the follow-up, the omission of multiple baseline measurements and the omission of long-term FU evaluations, which are typically included in more rigorously controlled case-series designs. The gold standard, naturally, aims to convey that a Randomized Controlled Trial (RCT) is the epitome of establishing the effectiveness of an intervention. When it comes to clinical practice, RCTs are not particularly beneficial in assessing the progress of individual clients. However, they can indeed prove useful in indicating which alternative treatments would be a promising starting point. On the other hand, psychotherapy studies are facing additional criticism for relying primarily on the average responses of large treatment groups, disregarding the variations within individuals that can influence the outcomes. In light of this criticism, researchers are now placing greater emphasis on the significance of individual treatment responses and the mechanisms of therapeutic change as the most effective means to improve efficacy (75). Single-case designs present an effective research methodology that systematically addresses individual differences over a period of time. This design significantly diminishes the likelihood of attributing changes solely to factors unrelated to the treatment and the conclusions drawn from such a study surpasses what can be derived from uncontrolled case studies (76). In the continued advancement of interventions for combat-related trauma, it is imperative for future research to build upon controlled case series and thoroughly examine the effectiveness of the intervention in addressing the wide array of symptoms commonly experienced by veterans.

## Data availability statement

The raw data supporting the conclusions of this article will be made available by the authors, without undue reservation.

## Ethics statement

The studies involving humans were approved by Medical Ethical Committee Amsterdam UMC. The studies were conducted in accordance with the local legislation and institutional requirements. The participants provided their written informed consent to participate in this study. Written informed consent was obtained from the individual(s) for the publication of any potentially identifiable images or data included in this article.

## Author contributions

MK: Investigation, Writing – original draft. MS: Investigation, Writing – original draft.

## Footnotes

^1^Prolonged Exposure (PE), Cognitive Processing Therapy (CPT), Cognitive Behavior Therapy (CBT) and Eye Movement Desensitization Reprocessing (EMDR).

^2^https://www.sensiks.com/. With the SENSIKS reality pod various war zones can be simulated.

^3^Video0 12 - YouTube for Afghanistan-deployed veterans, and sensiks forest final3 - YouTube for veterans who served in former Yugoslavia.

^4^See the documentary Nighttime in Kabul | A Cure For Fear: Part 2 | Topic - YouTube by Lana Wilson, in which a Canadian veteran was successfully treated using this approach.
